# The THINC-it Tool for Cognitive Assessment and Measurement in Major Depressive Disorder: Sensitivity to Change

**DOI:** 10.3389/fpsyt.2020.00546

**Published:** 2020-06-24

**Authors:** Roger S. McIntyre, Mehala Subramaniapillai, Caroline Park, Hannah Zuckerman, Bing Cao, Yena Lee, Michelle Iacobucci, Flora Nasri, Dominika Fus, Christopher R. Bowie, Tanya Tran, Joshua D. Rosenblat, Rodrigo B. Mansur

**Affiliations:** ^1^Mood Disorders Psychopharmacology Unit, University Health Network, Toronto, ON, Canada; ^2^Brain and Cognition Discovery Foundation, Toronto, ON, Canada; ^3^Department of Psychiatry, University of Toronto, Toronto, ON, Canada; ^4^Department of Pharmacology, University of Toronto, Toronto, ON, Canada; ^5^Institute of Medical Science, University of Toronto, Toronto, ON, Canada; ^6^Department of Psychology, Queen's University, Kingston, ON, Canada; ^7^Campbell Family Mental Health Research Institute, Centre for Addiction and Mental Health, Toronto, ON, Canada

**Keywords:** major depressive disorder, THINC-it tool, validation, screening, measurement, sensitivity

## Abstract

**Background:**

Herein, we sought to determine the sensitivity to change in cognitive function, as measured by the THINC-it tool, in a sample of adults with major depressive disorder (MDD) receiving standardized antidepressant therapy.

**Methods:**

Adults meeting the DSM-5 criteria for MDD with at least moderate depressive symptom severity [*i.e.*, Montgomery Åsberg Depression Rating Scale (MADRS) total score ≥ 20] were treated with open-label vortioxetine (10–20 mg/day, flexibly-dosed) for 8 weeks. The previously validated THINC-it tool was the primary dependent measure. The THINC-it tool was validated against the paper and pencil version of the Digit Symbol Substitution Test (DSST) and the Trails Making Test B (TMTB).

**Results:**

After 8 weeks of treatment, adults with MDD exhibited improvement in cognitive function relative to healthy controls (*e.g.*, processing speed) (p = 0.031). A subdomain measure of working memory (*i.e.*, symbol check; SC) exhibited significant improvement at Weeks 2 and 8 in latency (p = 0.032), SC accuracy (p = 0.046), and objective z-score (p = 0.001) independent of depressive symptoms. A linear regression analysis determined that the THINC-it tool measures of processing speed, as well as executive function were significantly associated with changes observed on the pencil and paper version the Digit Symbol Substitution Test (DSST) (p = 0.002) and in Trails Making Test B (TMTB) (p = 0.003), respectively.

**Conclusion:**

The THINC-it tool demonstrates sensitivity to change in adults with MDD and is highly correlated with improvements on pencil and paper versions of DSST and TMTB.

**Clinical Trial Registration:**

ClinicalTrials.gov, identifier NCT03053362.

## Introduction

Cognitive dysfunction in major depressive disorder (MDD) is identified as a predictor of functional outcome (1, 2). The clinical relevance of cognitive function in MDD provides the rationale and impetus for screening and assessment in clinical practice. Previously, our group validated the THINC-it tool in adults with MDD as an integrated, digitalized device to briefly assess cognitive functions at point of care (3). The THINC-it tool demonstrated sufficient psychometric properties in the clinical sample as well as in healthy volunteers.

Towards the aim of providing clinicians and researchers with a tool that is capable of not only screening for cognitive dysfunction, but also detecting sensitivity to change, a sensitivity to change validation study herein was conducted. The central aim of the study herein, was to evaluate sensitivity to change in cognitive function across repeat visits in adults with MDD. The clinical objective was to provide clinicians (and researchers) with a tool capable of being integrated into clinical practice to monitor cognitive function in adults with MDD.

## Methodology

### Participants

The primary study was an 8-week, open-label, sensitivity to change study assessing alterations in cognitive function/performance, as measured by the THINC-it tool (ClinicalTrials.gov Identifier: NCT03053362). The sample consisted of eligible adults (18–65 years of age) meeting the criteria for Diagnostic and Statistical Manual, Fifth Edition (DSM-5)-defined MDD who were treated with vortioxetine (10–20 mg flexibly dosed) (N = 100), and (N = 50) age, and sex-matched healthy controls (age-matched based on age categories). Vortioxetine was chosen as it is the only antidepressant recognized by the Food and Drug Administration (FDA) as having direct independent effects on cognition in adults with MDD. The 8-week period was selected due to the fact that this is a conventional window to look at change in acute studies. All subjects were enrolled at the Brain and Cognition Discovery Foundation (BCDF) in Toronto, ON. Prior to initiating the study, institutional review board consent was obtained, and all eligible participants provided written informed consent.

Participants met the following eligibility criteria: (1) provide written informed consent, (2) male or female 18–65 years of age, (3) a current diagnosis of a major depressive episode (MDE) as part of MDD as per DSM-5 criteria, (4) current MDE is confirmed by the Mini International Neuropsychiatric Interview (M.I.N.I 5.0.), (5) outpatient of a psychiatric setting, (6) MADRS score ≥ 20 at screening and baseline, (7) history of at least one prior MDE formally diagnosed by a healthcare provider or validated by previous treatment (*e.g.*, guideline-informed pharmacotherapy and/or manual-based psychotherapy) (4).

The exclusion criteria included: (1) current alcohol and/or substance use disorder as confirmed by the M.I.N.I 5.0, (2) presence of comorbid psychiatric disorder other than MDD that is a focus of clinical concern as confirmed by the M.I.N.I 5.0, (3) medications approved and/or employed off-label for cognitive dysfunction (*e.g.*, psychostimulants), (4) any medication for a general medical disorder that, in the opinion of the investigator, may affect cognitive function (*e.g.*, steroids, cholinomimetics, anticholinergics), (5) use of benzodiazepines within 12 h of cognitive assessments, (6) consumption of alcohol within 8 h of cognitive assessments, (7) inconsistent use or abuse of marijuana, (8) physical, cognitive, or language impairments sufficient to adversely affect data derived from cognitive assessments, (9) diagnosed reading disability or dyslexia, (10) clinically significant learning disorder by history, (11) electroconvulsive therapy (ECT) in the last 6 months, (12) history of moderate or severe head trauma (*e.g.*, loss of consciousness for >1 h), other neurological disorders, or unstable systemic medical diseases that, in the opinion of the investigator, are likely to affect the central nervous system, (13) pregnant and/or breastfeeding, (14) received investigational agents as part of a separate study within 30 days of the screening visit, (15) actively suicidal/presence of suicidal ideation or evaluated as being a suicide risk (as per clinical judgment using the Columbia-Suicide Severity Rating Scale), (16) currently receiving treatment with Monoamine Oxidase Inhibitors (MAOIs), antibiotics such as linezolid, or intravenous methylene blue, (17) previous hypersensitivity reaction to vortioxetine or any components of the formulation, (18) patients with angioedema, (19) clinical worsening symptoms of depression and suicide risk—in both adult and pediatric age groups, (20) serotonin syndrome, (21) abnormal bleeding, (22) previous history of mania/hypomania, (23) angle closure glaucoma, (24) hyponatremia, (25) moderate hepatic impairment (26), and history of seizures and epilepsy (4).

The eligibility criteria for the healthy controls were as follows: (1) no current or history of mental disorder as evidenced by the M.I.N.I. 5.0 for DSM-IV, (2) no first-degree relative with an established diagnosis by a healthcare provider of a mood or psychiatric disorder, (3) no unstable medical disorders. Healthy volunteers were excluded if any of the following criteria was met: (1) use of any medication for a general medical disorder and/or condition that, in the opinion of the investigator, may affect cognitive function (*e.g.*, corticosteroids, beta-blockers), (2) pregnant and/or breastfeeding, (3) consumption of alcohol within 8 hours of the THINC-it tool administration, (4) inconsistent use or abuse of marijuana (4).

### Clinical and Cognitive Assessment

The primary outcome measure in the study was sensitivity to change with the THINC-it tool (3). The THINC-it tool has been previously validated in adults with MDD (4). The THINC-it tool is digitalized and completed by the respondent on a tablet and includes variants of four objective, previously validated tests, Spotter (Choice Reaction Time), Symbol Check (1-back test), Trails (Trails Making Test B), and Codebreaker (Digit Symbol Substitution Test) as well as a self-reported cognitive function questionnaire (*i.e.*, Perceived Deficit Questionnaire, 5-item).

### Statistical Analysis

Between-group differences in baseline demographic characteristics were assessed using 2-sided chi-square, Mann–Whitney and t-tests. The intent-to-treat analysis included all randomized participants. For the longitudinal analysis, generalized estimating equation (GEE) models were used. Best fit was found with linear distributions and an independent covariance structure.

The independent variables included group (*i.e.*, HCs *vs* MDD), time (as a categorical variable), and group  ×  time interaction. Age, sex, and education were included as covariates. Associations between percentage change in tasks performance, adjusted for age and education, were assessed with Spearman's correlations. Finally, associations between changes in Codebreaker and Trails at Week 8 and changes in DSST and TMTB at Week 8, respectively, were assessed with linear regressions, adjusted for age, gender, education, and change in MADRS total score.

## Results

### Sample Description

One hundred participants with MDD (Mean age = 38.90, SD = 12.74) and 58 healthy volunteers (Mean age = 43.00, SD = 13.89) participated in testing at baseline. Participants' characteristics as well as baseline scores on clinical and cognitive measures are described in **Table 1**.

**Table 1:** Descriptive statistics of the ITT sample.

	Healthy Controls (n = 58)	MDD (n = 100)	p-value
Age (years), mean (SD)	43.00 (13.89)	38.90 (12.74)	0.067^a^
Sex (female), n (%)	27 (46.6)	67 (67.0)	0.012^b^
Years of education, mean (SD)	16.81 (2.96)	15.65 (3.06)	0.041^a^
Baseline MADRS, mean (SD)	1.64 (2.41)	31.83 (7.54)	<0.001^a^
Baseline DSST, mean (SD)	61.21 (14.25)	56.05 (14.70)	0.200^c^
Baseline TMTB, mean (SD)	70.59 (29.14)	72.94 (30.59)	0.559^a^
Baseline Spotter, mean (SD)	2.84 (0.11)	2.84 (0.13)	0.812^a^
Baseline SC latency, mean (SD)	3.01 (0.09)	3.02 (0.10)	0.524^a^
Baseline SC accuracy, mean (SD)	0.84 (0.33)	0.86 (0.30)	0.618^c^
Baseline Codebreaker, mean (SD)	54.33 (20.46)	57.54 (19.69)	0.385^a^
Baseline Trails, mean (SD)	37.36 (25.69)	33.48 (19.54)	0.482^a^
Baseline Objective z-score, mean (SD)	−0.12 (0.82)	−0.05 (0.76)	0.567^a^
Completers, n (%)	50 (86.20)	83 (83.00)	0.594^b^

^a^Mann–Whitney U; ^b^chi-square; ^c^t-test.

### Longitudinal Analysis

A longitudinal analysis was conducted while adjusting for age, gender, and education in both MDD and HC groups (**Table 2**). The main finding indicates that at both Weeks 2 and 8, performance of SC accuracy significantly improved in MDD participants compared to HCs (p = 0.031). Equal improvement in performance was observed in both groups for TMTB (p = 0.604), Spotter (p = 0.347), SC latency (p = 0.272), Codebreaker (p = 0.631), and in the objective z-score (p = 0.131). MDD participants improved slightly more than HCs in performance on DSST at Week 8 (p = 0.051). This result was also detected on Trails, while the performance for HCs on this task produced a J-shaped curve.

**Table 2:** P-values for time, group and time by group interaction from the longitudinal analysis, adjusted for age, gender and education.

	Time	Group	Time x Group	Comment
DSST	<0.001	0.006	0.051	Improving performance, slightly more pronounced in MDD, particularly at Week 8
TMTB	<0.001	0.247	0.604	Improving performance, equally in both groups
Spotter	<0.001	0.398	0.347	Improving performance, equally in both groups
SC latency	<0.001	0.299	0.272	Improving performance, equally in both groups
**SC accuracy**	**<0.001**	**0.128**	**0.031**	**Improving performance, significantly more pronounced in MDD at Week 2 and 8**
Codebreaker	<0.001	0.711	0.631	Improving performance, equally in both groups
Trails	<0.001	0.820	0.121	Improving performance in MDD, J-shaped curve in HC
Objective z-score	<0.001	0.948	0.131	Improving performance, equally in both groups

**Table 3** illustrates the longitudinal analysis conducted solely on MDD participants. When adjusting for age, gender, education, and MADRS score, significant improvements in SC latency (p = 0.032), SC accuracy (p = 0.046), and objective z-score (p = 0.001) were observed independent of depressive symptoms in Weeks 2 and 8. The Spotter task yielded similar results only in Week 2 (p = 0.005), and no improvement was detected for DSST, TMTB, Codebreaker, and Trails.

**Table 3:** P-values for time, group and time by group interaction from the longitudinal analysis, adjusted for age, gender, education, and MADRS score, in patients only.

	Time	P-Value	Comment
DSST	0.579	< 0.001	No improvement after adjustment for depressive symptoms
TMTB	0.604	0.004	No improvement after adjustment for depressive symptoms
Spotter	0.005	0.005	Significant improvement at Week 2, but not 8, independent of depressive symptoms
SC latency	< 0.001	0.032	Significant improvement at Week 2 and 8, independent of depressive symptoms
SC accuracy	< 0.001	0.046	Significant improvement at Week 2 and 8, independent of depressive symptoms
Codebreaker	0.541	0.003	No improvement after adjustment for depressive symptoms
Trails	0.720	0.003	No improvement after adjustment for depressive symptoms
Objective z-score	0.002	0.001	Significant improvement at Week 2 and 8, independent of depressive symptoms

### Correlation and Predictability of THINC-it

Correlations between the changes in performance of cognitive tasks were assessed for both Weeks 2 and 8. This is shown in **Table 4**. A significant correlation between the DSST and Codebreaker task was observed at Week 2 and Week 8 (p < 0.001). Additionally, at Week 8 we observed the following correlations: Spotter and DSST (p < 0.01); Trails and DSST (p < 0.05); and Trails and TMTB (p < 0.01). Age and level of education were adjusted in this analysis. These results show that the tasks in the THINC-it tool are associated with the standard pen and paper measures of cognition, which strengthens its potential to predict change in depressed populations.

**Table 4:** P-values for correlations between percentage change in tasks performance, adjusted for age and education.

Week 2		
	DSST	TMTB
Spotter	−0.073	0.092
SC latency	0.024	0.053
SC accuracy	0.080	−0.010
Codebreaker	0.422***	−0.031
Trails	−0.043	0.117
Objective z-score	−0.016	−0.021
Week 8		
	DSST	TMTB
Spotter	−0.279**	−0.027
SC latency	−0.167	−0.152
SC accuracy	0.118	−0.118
Codebreaker	0.353***	−0.163
Trails	−0.211*	0.229**
Objective z-score	0.006	0.034

*p < 0.05; **p < 0.01; ***p < 0.001.

A linear regression analysis was conducted to determine the predictability of the THINC-it tool at Week 8 (**Table 5**). The THINC-it tasks that generated these results were Codebreaker and Trails. The changes in Codebreaker and Trails were largely predicted by changes in DSST (p = 0.002) and in TMTB (p = 0.003), respectively.

**Table 5:** Linear regressions, change in Codebreaker and Trails at Week 8, predicted by change in DSST and TMTB at Week 8, respectively, adjusted for age, gender, education and change in MADRS, in patients only.

Change in Codebreaker	Beta	p-value	R^2^ = 0.170, p = 0.015
Change in DSST	0.715	0.002	
Change in MADRS	0.073	0.562	
Age	0.003	0.268	
Gender	−0.053	0.498	
Education	−0.014	0.269	
Change in Trails	Beta	p-value	R^2^ = 0.127, p = 0.064
Change in TMTB	0.338	0.003	
Change in MADRS	0.077	0.442	
Age	0.001	0.577	
Gender	−0.014	0.818	
Education	−0.003	0.729	

## Discussion

Herein, the THINC-it tool demonstrates sensitivity to change consistent with previously validated standardized paper and pencil neuropsychological tasks. Our results extend the original validation results indicating that the THINC-it tool may be used as both a screening and repeat measures tool in adults with MDD. A recent expert panel provided recommendations for including cognitive measures as part of routine assessment of care in adults with MDD (5). Future research vistas for the THINC-it tool are to determine whether implementation of the tool in clinical practice improves therapeutic outcomes in depression and/or is cost-effective.

Strengths of this sensitivity to change analysis are the representativeness of the patients (*e.g.*, minimal exclusion criteria, the allowance of previous and concomitant medications, and individuals who have both psychiatric and medical comorbidity). A related strength is that the medication chosen (*i.e.*, vortioxetine) is recognized by the US FDA for having an independent and direct effect on cognitive function in individuals with MDD (6).

A limitation of our study that may affect the interpretations of the results include the notion that individuals who consented to treatment (*i.e.*, agreed to be a research participant) are not representative of all patients with MDD. Additionally, although we have shown that the THINC-it tool can be used for interventional studies in adults with MDD wherein the outcome measure of interest is cognition, further research is needed to extend its use to other types of studies. It is also not determined whether the THINC-it tool would be sensitive to change in individuals with cognitive dysfunction treated for longer periods of time (*i.e.*, beyond eight weeks of treatment). Additionally, given that our MDD sample had an overall cognitive performance at the group level similar to healthy controls at the outset, extrapolating these results to other subpopulations would need to be studied empirically. However, what is most pertinent about this study is examining the sensitivity of the THINC-it tool to change within the group across repeat measures. Taken together, the THINC-it tool demonstrates sensitivity to change in this study. The brevity of the scale, its self-administration, and its point of care capability are all facilitators of implementation in the clinical setting. The THINC-it tool may also be conceptualized as a dependent measure of cognitive function in the research ecosystem (6, 7). Future studies should also compare the THINC-it tool to other existing cognitive measures to determine which scales are capable of enhancing assay sensitivity in clinical trials designed to evaluate cognitive outcomes in MDD.

In summary, the THINC-it tool, previously validated as a screening tool for cognition in MDD, demonstrates sensitivity to change in a cohort of adults receiving care for MDD. Measurement-based care has been demonstrated to improve health outcomes in MDD; whether integrating the THINC-it tool into measurement-based tools in MDD augments therapeutic outcomes and/or is cost-effective is the priority next step research question.

## Accessing the THINC-IT Tool

The THINC-it tool is a digital tool that can be downloaded for free from the following website: https://progress.im/en/content/download-thinc-it%C2%AE-tool

## Data Availability Statement

The raw data supporting the conclusions of this article will be made available by the authors, without undue reservation.

## Ethics Statement

The studies involving human participants were reviewed and approved by REB—REB (Community). The patients/participants provided their written informed consent to participate in this study.

## Author Contributions

RM contributed to the conceptualization and writing of the original draft. CB and TT contributed to the data analysis, writing, review, and editing. All authors contributed to the article and approved the submitted version.

## Funding

This research study was supported by an investigator-initiated unrestricted grant to the Brain and Cognition Discovery Foundation by Lundbeck, Denmark. The sponsor played no role in the design, execution, data analytics or manuscript preparation of this study.

## Conflict of Interest

RM has received research grant support from Stanley Medical Research Institute, CIHR/GACD/National Natural Science Foundation of China; speaker/consultation fees from Lundbeck, Janssen, Shire, Purdue, Pfizer, Otsuka, Allergan, Takeda, Neurocrine, Sunovion, Minerva. MS has received an honoraria for publication of a review article unrelated to the current article by Institut La Conference Hippocrate (AICH). CB has received grant support from Lundbeck, Pfizer, and Takeda. He is a consultant for Boehringer Ingelhiem, Lundbeck, and Pfizer. JR has received research grant support from the Canadian Cancer Society, Canadian Psychiatric Association, American Psychiatric Association, American Society of Psychopharmacology, University of Toronto, University Health Network Centre for Mental Health, Joseph M. West Family Memorial Fund and Timeposters Fellowship and industry funding for speaker/consultation/research fees from Allergan, Lundbeck and COMPASS. JR is the medical director of a private clinic providing off-label ketamine infusions for depression.

The remaining authors declare that the research was conducted in the absence of any commercial or financial relationships that could be construed as a potential conflict of interest.
